# Analysis of Human TAAR8 and Murine Taar8b Mediated Signaling Pathways and Expression Profile

**DOI:** 10.3390/ijms151120638

**Published:** 2014-11-10

**Authors:** Jessica Mühlhaus, Juliane Dinter, Daniela Nürnberg, Maren Rehders, Maren Depke, Janine Golchert, Georg Homuth, Chun-Xia Yi, Silke Morin, Josef Köhrle, Klaudia Brix, Matthias Tschöp, Gunnar Kleinau, Heike Biebermann

**Affiliations:** 1 Institut für Experimentelle Pädiatrische Endokrinologie, Charité-Universitätsmedizin, Campus Virchow-Klinikum, Augustenburger Platz 1, 13353 Berlin, Germany; E-Mails: jessica.muehlhaus@charite.de (J.M.); juliane.dinter@charite.de (J.D.); daniela.nuernberg@charite.de (D.N.); gunnar.kleinau@charite.de (G.K.); 2 School of Engineering and Science, Research Center MOLIFE—Molecular Life Science, Jacobs University Bremen, Campus Ring 1, 28759 Bremen, Germany; E-Mails: m.rehders@jacobs-university.de (M.R.); k.brix@jacobs-university.de (K.B.); 3 Interfaculty Institute for Genetics and Functional Genomics, University Medicine and Ernst-Moritz-Arndt-University Greifswald, Friedrich-Ludwig-Jahn-Str. 15a, 17487 Greifswald, Germany; E-Mails: depke@uni-greifswald.de (M.D.); janinegolchert@web.de (J.G.); georg.homuth@uni-greifswald.de (G.H.); 4 Helmholtz Zentrum München, German Research Center for Environmental Health, Institute for Diabetes and Obesity, Business Campus Garching, Parkring 13, 85748 Garching, Germany; E-Mails: chun-xia.yi@helmholtz-muenchen.de (C.-X.Y.); silke.morin@helmholtz-muenchen.de (S.M.); matthias.tschoep@helmholtz-muenchen.de (M.T.); 5 Institut für Experimentelle Endokrinologie, Charité-Universitätsmedizin Campus Virchow-Klinikum, Augustenburger Platz 1, 13353 Berlin, Germany; E-Mail: josef.koehrle@charite.de; 6 Division of Metabolic Diseases, School of Medicine, Technische Universität München, Schneckenburgerstraße 8, 81675 München, Germany

**Keywords:** trace amine-associated receptor, TAAR8, 3-T_1_AM, thyronamine, signaling pathways, basal activity

## Abstract

The thyroid hormone derivative 3-iodothyronamine (3-T_1_AM) exerts metabolic effects *in vivo* that contradict known effects of thyroid hormones. 3-T_1_AM acts as a trace amine-associated receptor 1 (TAAR1) agonist and activates G_s_ signaling *in vitro*. Interestingly, 3-T_1_AM-meditated *in vivo* effects persist in *Taar1* knockout-mice indicating that further targets of 3-T_1_AM might exist. Here, we investigated another member of the TAAR family, the only scarcely studied mouse and human trace-amine-associated receptor 8 (Taar8b, TAAR8). By RT-qPCR and locked-nucleic-acid (LNA) *in situ* hybridization, *Taar8b* expression in different mouse tissues was analyzed. Functionally, we characterized TAAR8 and Taar8b with regard to cell surface expression and signaling via different G-protein-mediated pathways. Cell surface expression was verified by ELISA, and cAMP accumulation was quantified by AlphaScreen for detection of G_s_ and/or G_i/o_ signaling. Activation of G-proteins G_q/11_ and G_12/13_ was analyzed by reporter gene assays. Expression analyses revealed at most marginal *Taar8b* expression and no gender differences for almost all analyzed tissues. In heart, LNA-*in situ* hybridization demonstrated the absence of *Taar8b* expression. We could not identify 3-T_1_AM as a ligand for TAAR8 and Taar8b, but both receptors were characterized by a basal G_i/o_ signaling activity, a so far unknown signaling pathway for TAARs.

## 1. Introduction

Trace amine-associated receptors (TAAR) belong to family A of G protein-coupled receptors (GPCR) [1]. TAARs are evolutionarily conserved throughout diverse vertebrates, including humans (six TAARs, three pseudogenes) [2,3,4,5]. So far as is known, the predominant signaling pathway for TAARs utilizes G_s_ activation [6,7,8,9,10] or activation of G_olf_ [7]. TAARs are ubiquitously expressed in humans and rodents, with expression in various brain regions, as well as in kidney, stomach, liver, pancreas, small intestine, pituitary, and leukocytes [11,12,13,14,15,16]. In addition, a particularly high expression of several TAARs, with the exception of TAAR1, was reported in the olfactory system of vertebrates [7,8,17,18,19,20,21].

The thyroid hormone derivative 3-iodothyronamine (3-T_1_AM) was described in 2004 as a potent agonist of TAAR1, stimulating cAMP production in *in vitro* experiments [9]. Moreover, *in vivo* administration of 3-T_1_AM in mice induced hypothermia and bradycardia [9]. On isolated rat hearts, application of 3-T_1_AM resulted in reduced heart rates [9,10]. Collectively, these results highlighted possible cardio-protective effects and suggested that 3-T_1_AM might also protect from major brain damage upon stroke [22,23]. However, *Taar1*-deficient mice (*Taar1*^−/−^) showed no significant differences regarding their general health status, and the hypothermic effect of 3-T_1_AM was even reproduced in *Taar1*^−/−^ mice, pointing to a different mediator of 3-T_1_AM action *in vivo* [24,25].

Given the reported effects of 3-T_1_AM in wild type and *Taar1*^−/−^ mice for which the mediator remains unknown (see above), we speculated and tested TAAR8 orthologues as putative targets for 3-T_1_AM action. To date, the known signaling pathway for TAARs is mediated via G_s_ activation [6,7,26], but the reported effects of 3-T_1_AM on the heart should be comprehensively explained by G_i/o_ coupling. We therefore aimed to find a TAAR subtype which potentially activates G_i/o_ and not G_s_. As the *in vivo* effects of 3-T_1_AM have primarily been studied in mice [9,10,25], we focused on the poorly characterized human TAAR8 and its murine orthologue *Taar8b*. We investigated its expression pattern in different tissues and performed a thorough *in vitro* analysis of the signal transduction properties of Taar8b in comparison with the human TAAR8.

## 2. Results

Here we focus on human and mouse trace amine-associated receptor 8 orthologues as possible targets of 3-T_1_AM action. We analyzed putatively involved G protein-dependent signaling pathways, *i.e.*, G_s_, G_i/o_, G_q/11_, and G_12/13_, as triggered by signaling via the murine and human orthologues at basal conditions and following stimulation with known agonists of TAAR1. Additionally, we investigated the expression of *Taar8b* in various mouse tissues.

### 2.1. The Murine Taar8b Is at Most Marginally Expressed in Several Mouse Tissues

**Figure 1:**
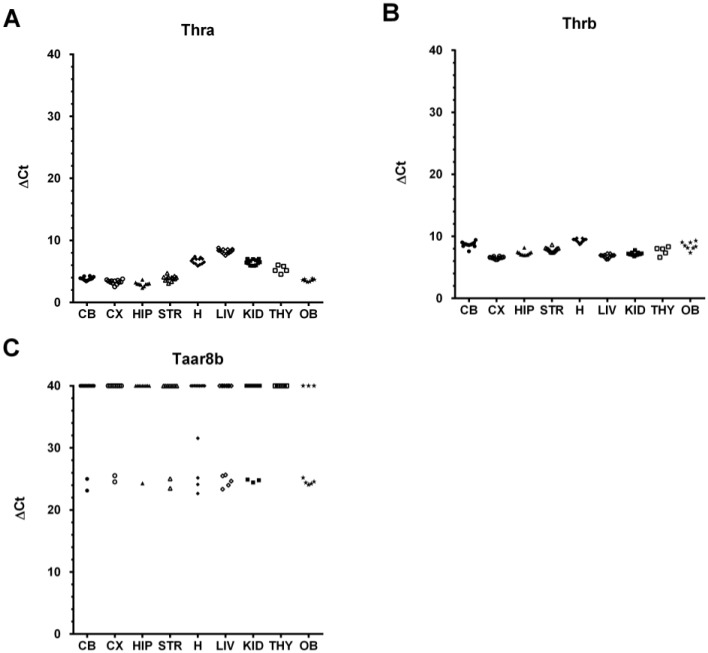
Normalized expression levels of the positive control genes *Thra* and *Thrb* and the target gene *Taar8b*. Normalization was performed by Δ*C*_t_-value calculation, *i.e.*, by subtracting the *C*_t_-value of the calibrator gene *Gapdh* from the *C*_t_-value of the positive control or target gene (Δ*C*_t,_(*Thra*) = *C*_t_(*Thra*) – *C*_t_(*Gapdh*); Δ*C*_t_(*Thrb*) = *C*_t_(*Thrb*) – *C*_t_(*Gapdh*); Δ*C*_t,*Taar8b*_ = *C*_t_(*Taar8b*) – *C*_t_(*Gapdh*)). The Δ*C*_t_-values of samples which yielded “undetermined” *C*_t_-values in PCR runs of 50 cycles for all technical replicates were artificially set to 40 and, thus, are visible above the group of samples with detectable *C*_t_-values (*C*_t_ < 50). In all other cases, Δ*C*_t_-values were calculated from mean *C*_t_-values of the technical replicates for which a *C*_t_-value was measured. If only one technical replicate yielded a *C*_t_-value smaller than 50, this *C*_t_-value was used for Δ*C*_t_-calculation. Positive control genes *Thra* and *Thrb* were not included in the analysis of three thyroid samples because of the limited amount of RNA. Graphs are depicted for the positive control genes *Thra* (**A**) and *Thrb* (**B**) and for the target gene *Taar8b* (**C**). Samples from male and female mice for each tissue type are collectively grouped. CB, cerebellum; CX, cortex; HIP, hippocampus; STR, striatum; H, heart; LIV, liver; KID, kidney; THY, thyroid; OB, olfactory bulb; *C*_t_, threshold cycle; *Gapdh*, glyceraldehyde-3-phosphate dehydrogenase; *Thra*, thyroid hormone receptor alpha; *Thrb*, thyroid hormone receptor beta; *Taar8b*, trace amine-associated receptor 8B.

In a previous study performed by Liberles and co-workers, marginal levels of *Taar8b* expression in the olfactory epithelium were detected [7]. We used RT-qPCR to analyze further tissue expression of murine *Taar8b*. For this purpose, we first verified the absence of contamination of the RNA preparations with residual DNA traces for all samples. While the expression levels of the reference gene *Gapdh* as well as the positive controls *Thra* and *Thrb* were comparable between the individual animals and detected to be rather high for *Gapdh* (*C*_t_-value average 18.75) and lower for *Thra* and *Thrb* (*C*_t_-value averages 23.68 and 26.38), respectively, *Taar8b* expression was observed to be considerably lower (*C*_t_-values ranging from 41.21 to 49.14, average 42.95). Clear differences were not generally detected between male and female animals. Low expression is commonly known to be reflected in a higher variation of the *C*_t_-values between biological and technical RT-qPCR replicates. This effect was also evident in our sample set (Figure 1 and Table S1). We measured detectable *C*_t_-values (<50) for one, two, or three of the technical replicates, but also observed no detectable *C*_t_-values in all technical replicates per individual tissue sample in our qPCR setting which was comprised of 50 cycles of amplification (Table S1). *C*_t_-values less than 50 in at least one technical replicate were only observed for 24 of 101 individual tissue samples analyzed (Figure 1), thereby missing our standard criteria for validated expression (at least two comparable technical replicates in at least three biological replicates). The expression level of *Taar8b* was at most marginal and close to the detection limit. *Taar8b* transcripts were not detected in thyroid tissue via qPCR (50 cycles).

**Figure 2:**
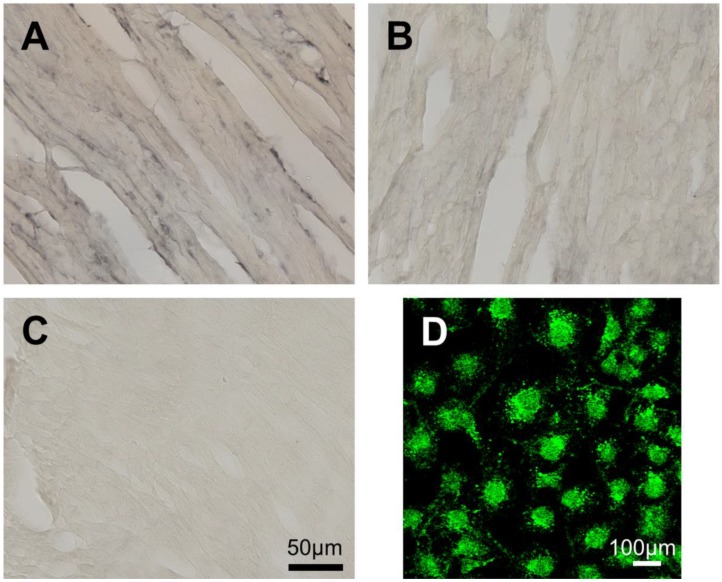
Expression studies of Taar8b in murine heart using *in situ* hybridization. Expression studies were performed by *in situ* hybridization (ISH) using a LNA (locked nucleic acid) probe. C57BL/6 WT mouse hearts were sectioned and treated with the LNA probe. Signals were visualized with an avidin–biotin complex using DAB (3,3'-diaminobenzidin) (**A**–**C**) or DY-light 488 streptavidin (**D**) staining. (**A**) The *mGata* probe used as a positive control caused significant staining of the nucleus and the perinuclear region of cardiomyocytes, as expected; (**B**) The negative control performed by hybridization with scrambled ISH probe demonstrated homogenous shading; (**C**) Treatment with *Taar8b* specific probes did not result in staining of the heart tissue, pointing to a lack of expression of *Taar8b* in the murine heart; (**D**) COS-7 cells transiently transfected with *Taar8b* served as a positive control for the *Taar8b* probe, exhibiting prominent DyLight-488 positive signal , thereby proving functionality of the probe.

As it is known that TAARs, in general, are expressed at low levels except for in the olfactory epithelium [7,26], we applied the very sensitive method “locked nucleic acid” (LNA) *in situ* hybridization (ISH) for the example of heart tissue. In this particular case, we were able to demonstrate the absence of *Taar8b* mRNA for this tissue (Figure 2).

### 2.2. Cell Surface Expression of TAAR8

**Figure 3:**
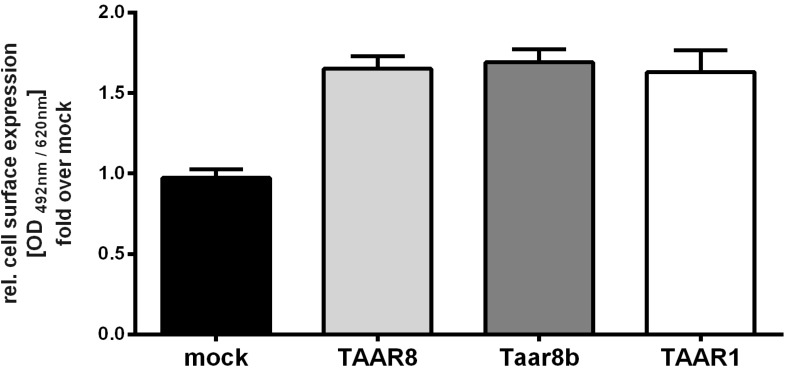
Cell surface expression of TAAR8. Cell surface expression was determined in COS-7 cells by ELISA as described in the methods section. Data are represented as mean ± SEM fold over mock (0.02 ± 0.001) (black) relative cell surface expression, measured at OD_492/620 nm_ and based on three to six independent experiments with six replicates each. TAAR8 (light grey), Taar8b (dark grey) and human TAAR1 (white) showed comparable surface expression. The human β2-adrenergic receptor (ADRB2) served as a positive control, demonstrating expression levels of 23.72 ± 1.59 (mean OD_492/620 nm_ ± SEM, fold over basal mock).

In order to exclude lack of receptor signaling due to possible misfolding and/or premature retention of the receptor, we performed cell surface expression studies by ELISA with the *N*-terminally HA-tagged receptor constructs. The receptor orthologues TAAR8 and Taar8b demonstrated low but robust cell surface expression in comparison to the empty vector (mock, negative control) when expressed in COS-7 cells (Figure 3). The human β2-adrenergic receptor (ADRB2) served as a positive control which showed expression levels of 23.72 ± 1.59 (mean OD_492/620 nm_ ± SEM fold over mock).

### 2.3. Identification of Basal G_i/o_ Mediated Signaling

**Figure 4:**
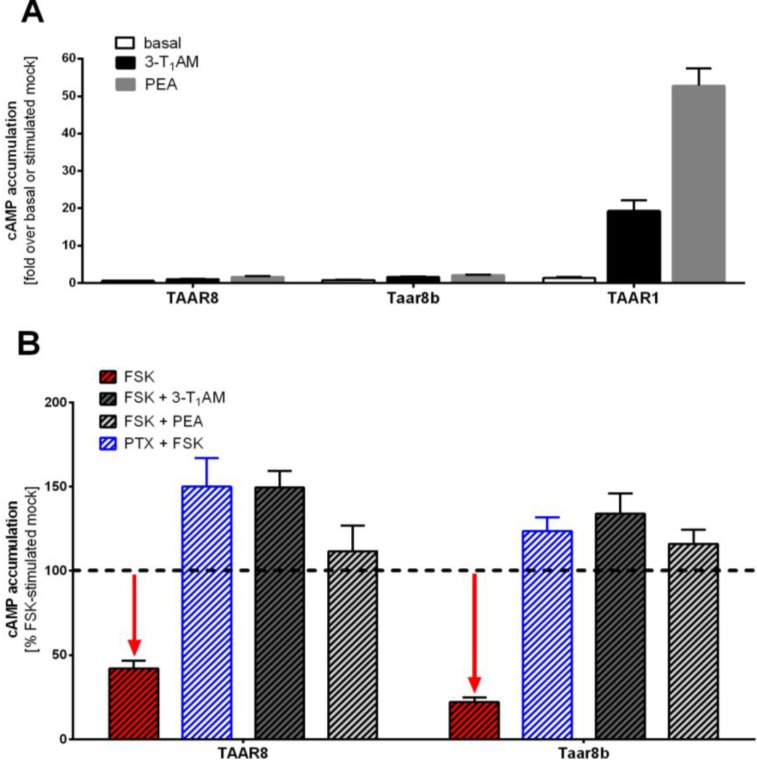
cAMP accumulation after G_s_ and G_i/o_ coupling, respectively. HEK293 cells were stimulated with either 3-T_1_AM (black) or PEA (grey) for determination of potential G_s_ signaling (**A**) or co-stimulated with forskolin (FSK) and 3-T_1_AM (dark grey, hatched), PEA (light grey, hatched) or pertussis toxin (PTX) (blue hatched), for determination of potential G_i/o_ signaling (**B**). Accumulation of cAMP was determined by AlphaScreen technology as described in the methods section. Data are represented as mean ± SEM of cAMP accumulation in fold over basal or stimulated mock-treated cells, based on two to seven independent experiments, each performed in triplicate. (**A**) Mock-transfected cells showed basal cAMP levels of 0.71 ± 0.13 nM. The human TAAR1 served as a positive control; (**B**) Mock-transfected cells demonstrated basal cAMP levels of 18.5 ± 1.1 nM. Murine and human TAAR8 showed basal G_i/o_ coupling activity, indicated by a low basal cAMP accumulation compared to mock transfected cells (dotted line = mock 100% baseline) and by increased cAMP accumulation following pre-incubation with PTX and stimulation with FSK.

The accumulation of cAMP was determined under basal conditions for TAAR8 and Taar8b (Figure 4) in HEK293 cells. Human TAAR1 and the human 5-hydroxytryptamine (serotonin) receptor 1B (5HT_1B_) served as positive controls (not shown). Cells transiently expressing Taar8b, TAAR8, TAAR1 or 5HT_1B_ showed no cAMP accumulation (Figure 4A). These levels remained below the endogenous cAMP levels of mock-transfected cells.

Application of 10 µM 3-T_1_AM or 10 µM β-phenylethylamine (PEA) did not result in an increase in cAMP accumulation for TAAR8 or Taar8b in comparison to cells expressing TAAR1 (Figure 4A).

Furthermore, we prestimulated transfected cells with forskolin (FSK) to induce an overall activation of the adenylyl cyclase. Indeed, cAMP levels of cells expressing the murine and human TAAR8 showed a reduction of 58% (TAAR8) and 78% (Taar8b), compared to FSK-stimulated mock transfected cells (Figure 4B), indicating strong constitutive G_i/o_ activity. To confirm G_i/o_ activation, we pre-incubated the cells with pertussis toxin (PTX), which catalyzes ADP-ribosylation of G_i/o_, preventing the G_i/o_ protein from interacting with GPCR, which led to a significant increase in cAMP accumulation up to the levels of mock-transfected cells under FSK stimulation. These results strengthened the finding of a G_i/o_ basal activity of TAAR8 and Taar8b orthologue (Figure 4B).

### 2.4. G_12/13_ or G_q/11_ Signaling Pathways

Finally, we examined the activation of G protein-coupling pathways G_12/13_ and G_q/11_ under basal and ligand-incubated conditions (Table 1). For activation of the G_12/13_ pathway, no significant receptor signaling was detected, regardless of whether basal or 3-T_1_AM or PEA-stimulated conditions were analyzed. Additionally, we found a marginal increase in IP3 formation (related to G_q/11_ activation) for TAAR8 under basal conditions, which should also be attributed to the low signaling activity of the β/γ subunits of G_i/o_. However, this was not observed for HEK293 cells expressing Taar8b.

**Table 1:** Basal signaling activity and stimulation of G_q/11_ and G_12/13_ pathways with 3-T_1_AM and PEA at TAAR8 and Taar8b. Cells over-expressing TAAR8 and Taar8b were stimulated with either 10 µM 3-T_1_AM or 10 µM PEA. Values represent luciferase activities (IP3–luc, RhoA–luc) and are indicated as mean ± SEM relative light units (rlu) in fold over basal or ligand-stimulated mock, each based on three to five independent experiments performed in triplicate. Mock transfected cells showed basal levels of 5003 ± 930 rlu for IP3-luc and 209,855 ± 27,087 rlu for RhoA-luc. The human thyrotropin receptor (TSHR) served as a positive control. Cells transfected with TSHR demonstrated basal levels of 3442 ± 589 rlu (IP3–luc) or 435,437 ± 70,197 rlu (RhoA–luc). Stimulation of TSHR was found to be within a range of 64.4 ± 13.0 rlu (IP3–luc) or 17.4 ± 4.7 rlu (RhoA–luc) fold over basal mock.

TAAR Subtype	Second Messenger Signaling	Ligand	Basal Activity (Fold Over Basal Mock)	Ligand Stimulated (Fold Over Stimulated Mock)
TAAR8	IP3–luc	3-T_1_AM	1.6 ± 0.1	1.1 ± 0.2
		PEA		1.1 ± 0.2
	RhoA–luc	3-T_1_AM	1.2 ± 0.1	1.5 ± 0.3
		PEA		1.2 ± 0.1
Taar8b	IP3–luc	3-T_1_AM	1.2 ± 0.1	0.9 ± 0.1
		PEA		1.4 ± 0.2
	RhoA–luc	3-T_1_AM	1.0 ± 0.1	0.8 ± 0.1
		PEA		0.9 ± 0.1

## 3. Discussion

The objective of the present study was to characterize the signaling properties of TAAR8 and Taar8b, also following challenge with potential ligands. PEA and 3-T_1_AM were used for stimulation approaches as both are known agonists for TAAR1 [9,25]. Additionally, we analyzed the expression profile of *Taar8b* in mice.

Low expression of *Taar8b* has been previously described for the olfactory epithelium [7]. We describe herein that expression of *Taar8b* in young male and female C57BL6/J mice is at most marginal and close to the detection limit in the analyzed tissues. Detection was impeded by high variation due to the very low transcript levels and was only possible for a fraction of each sample set (Figure 1 and Table S1). We measured detectable *C*_t_-values in at least one technical replicate for 24 of 101 analyzed samples, independent from the tissue type. In our experiments, *Taar8b* expression was not detectable in the thyroid tissue of C57BL6/J mice (Figure 1 and Table S1). It has to be concluded that expression levels of *Taar8b* were at most marginal and very close to the detection limit. Finally, a clear non-ambiguous assessment of the expression levels in the analyzed tissues was not possible based on the RT-qPCR results.

As expression of *Taar8b* has been described to be undetectable in the heart tissue of C57BL6/J mice, an observation in line with others [27], we additionally applied the very sensitive LNA *in situ* hybridization for this tissue. We confirmed the published findings (Figure 2). Hence, from our data and those of others, we conclude that the negative inotropic effects of 3-T_1_AM administration in WT and *Taar1* knockout mice are highly unlikely to be mediated by Taar8b activation in the mouse heart. Further studies are required to elucidate the mediator of 3-T_1_AM effects. This is particularly important as 3-T_1_AM has been proposed as a potential neuroprotective compound for stroke patients [23]. If it targets other ubiquitously expressed receptors, this may lead to further, thus far unexpected effects in other tissues.

Recent findings by D’Andrea and colleagues which demonstrate that *TAAR8* transcripts in astroglial cells are up-regulated following stimulation with lipo-polysaccharides strengthened the assumption of a distinct functionality in the brain [28]. However, *Taar8b* transcripts appear to be scarce in brain tissue, if present at all [29]. Likewise, in the RNA-Seq-based *Illumina Body Map*, *TAAR8* expression was not detected in human brain tissue. To set this into context, it has to be emphasized that the corresponding *Body Map* RNA-Seq analysis was performed with total RNA prepared from whole human brain [30]. Thus, even if expression of *TAAR8* would occur in detectable amounts in defined sub-regions of the brain, it is unlikely that transcript levels are abundant enough to be detected if whole-brain RNA instead of RNA prepared from these defined sub-regions is used. However, the functional implications of the regionally restricted expression of *Taar8b* in murine liver and specific brain regions require further investigation.

We detected basal G_i/o_ activity for the murine Taar8b and human TAAR8, which *per se* does not exclude the activation of other pathways by ligand interaction or in different cell-types as used here. A simultaneous activation of two different signaling pathways is a known concept for GPCRs [31]. The α2C-adrenergic receptor, for instance, is capable of coupling to both, G_s_ and G_i/o_, [32]. Likewise, the β2-adrenergic receptor in cardiomyocytes also couples to both, G_s_ and G_i/o_ [33]. In this context, it is important to remember that specificity for a certain G protein is determined by several parameters such as (i) agonist concentration or type, (ii) expression levels of the receptor [32,34], (iii) cell type [35,36], and (vi) the receptor amino acid constitution (intermolecular interaction pattern) [35]. Nevertheless, the basal level of G_i/o_-mediated signaling found here for Taar8b and TAAR8 point to a permanent inhibitory regulation.

The increase of cAMP accumulation levels following stimulation with 3-T_1_AM in FSK pre-incubated cells appears inconsistent regarding the fact that 3-T_1_AM alone does not significantly activate G_s_ signaling pathway. However, we could not exclude that TAAR8 forms heterodimers with other receptors (reviewed in [37]), which might promote a different signaling profile and response to 3-T_1_AM. In addition, although we do not yet know the exact endogenous ligand of TAAR8, one should consider that a ligand-independent regulating receptor-function is feasible [38]. These functions may be the capacity to modulate receptor folding, cell surface expression, and signaling of other GPCRs by forming heterodimers. Such a scenario was observed for the non-orphan GABA_B1_ receptor that requires the orphan GABA_B2_ receptor for cell surface expression rendering both fully functional [39,40,41]. The level of orphan receptor expression in relation to that of its non-orphan receptor partner might serve as a regulatory mechanism, as was described for heterodimerization of the orphan GPR50 with the non-orphan melatonin receptors 1, which leads to inhibition of signaling function [38].

## 4. Experimental Section

### 4.1. Expression Analysis of Taar8b in the Heart by in Situ Hybridization

Murine *Taar8b* was analyzed for potential expression in the mouse heart by *in situ* hybridization histochemistry using digoxigenin (DIG)-labeled locked nucleic acid (LNA) probes (Exiqon Inc., Woburn, MA, USA). All studies were approved by and performed in accordance to the guidelines of the Institutional Animal Care and Use Committee (IACUC) of the University of Cincinnati (Cincinnati, OH, USA; Protocol number 06-08-07-01; approved 28 January 2010).

Wild type (WT) C57BL/6 mice were sacrificed by an intra-atrial perfusion with saline, followed by a solution of 4% paraformaldehyde in 0.1 M phosphate buffer (pH 7.4) at 4 °C. Hearts were then isolated and post-fixed in 4% paraformaldehyde for 16 h. Following equilibration for 48 h in RNase free 30% sucrose in 0.1 M Tris-buffered saline, tissue was cut into 25 μm sections.

Sections of heart were washed successively with PBS, 0.2 M HCl, and incubated in 0.2% glycine followed by 0.1% Triton X-100. Free floating sections were then pre-hybridized in 1× pre-hybridization buffer and 50% formamide (Sigma–Aldrich, St. Louis, MO, USA) for 1 h at 55 °C on a rocking platform. For hybridization, heart sections were incubated for 8 h in 200 nM LNA probes containing hybridization buffer (Sigma–Aldrich) at 57 °C. After stringent washing steps with decreasing concentrations of saline–sodium citrate, samples were incubated with 1:500 diluted anti-DIG antibody (goat) at 4 °C overnight. In a next step, samples were washed with Tris–Borat–EDTA-buffer (TBE) and incubated with an avidin–biotin–peroxidase complex (ABC) for 1 h at room temperature. For visualization, heart sections were stained with 3,3'-diaminobenzidin (DAB) for 5 min. Sections were mounted on gelatin-coated glass slides, dried, dehydrated through a graded ethanol series, cleared in xylene and cover-slipped for image collection by light microscopy.

A *Gata4* probe served as a positive control for *in situ* hybridization. To test the functionality of the *Taar8b* probe, COS-7 cells were transiently transfected with *Taar8b* and hybridized as described for the heart sections. For visualization, COS-7 cells were stained with anti-DIG antibody as described above, followed by a Dy-Light-488 conjugated secondary anti-goat IgG (Jackson ImmunoResearch Laboratories, West Grove, PA, USA). Images were collected by confocal microscopy (Zeiss confocal 710, Carl Zeiss Microscopy GmbH, Jena, Germany).

### 4.2. Expression Analysis of Taar8b by RT-qPCR

Housing and breeding of WT C57BL6/J mice were conducted in accordance with institutional guidelines at the S1-laboratories of Jacobs University, Bremen, Germany, registered as SfAFGJS Az. 513-30-00/2-15-32 and 522-27-11/3-1, 05-A20 and A21. Mice were housed under standard conditions, with a 12 h/12h light/dark cycle with lights on from 07:00 to 19:00, and *ad libitum* water and food.

Heart, liver, kidney, thyroid and brain tissue from different regions were prepared from young male and female mice. The animals were anesthetized with IsoFlo^®^ (CP Pharma Handelsgesellschaft GmbH, Burgdorf, Germany), followed by i.p. injection of ketamine (Bela-pharm, Vechta, Germany). The abdominal and thoracic cavities were opened and the abdominal aorta was cut. Perfusion was performed through the left ventricle with 200 IU heparin (B. Braun Melsungen AG, Melsungen, Germany) in 0.9% NaCl solution. Tissue specimens of interest were immediately snap-frozen in liquid nitrogen after preparation and subsequently stored at −80 °C.

Tissue samples were first disrupted into smaller pieces under liquid nitrogen using a mortar and pestle. Sample preparation was continued with a tissue aliquot of suitable size. Tissue specimen homogenization combined with simultaneous RNA extraction was performed using a modification of the Trizol reagent-based extraction method described by Chomczynski and Sacchi [42]. First, tissue samples and 500 µL Trizol reagent (Invitrogen/Life Technologies, Karlsruhe, Germany) were transferred to disruption Teflon vessels containing a disruption ball, where the complete system was pre-cooled in liquid N_2_. Subsequently, the closed vessel was inserted into a bead mill dismembrator (Braun, Melsungen, Germany), and sample disruption was performed at 2600 rpm for 2 min. An additional 500 µL of Trizol reagent was added to the frozen tissue powder. Following thawing, 1 mL total volume of total RNA-containing TRIzol solution was removed and further processed. The final RNA preparations were additionally DNase-treated (RNase-Free DNase Set, Qiagen, Hilden, Germany) and purified using a Norgen RNA Clean-Up and Concentration Micro Kit (Norgen, Biotech Corp., Thorold, ON, Canada). Concentration and purity of RNA preparations were determined using a ND-8000 spectrophotometer (Thermo Fisher Scientific Inc., Wilmington, DE, USA), and RNA integrity was validated by means of lab-on-a-chip capillary electrophoresis technology using an Agilent 2100 Bioanalyzer (Agilent Technologies, Santa Clara, CA, USA). RNA quality control values are available as Table S2. In order to control for residual contaminations of genomic DNA in the RNA preparations, no-amplification-control-reactions (NACs) were performed for all samples and targeted genes. Here, qPCR reactions were assembled where the usual cDNA was exchanged with the corresponding amount of RNA. Samples yielding positive signals in any NAC-reaction (*C*_t_ < 50) were subjected to an additional DNase-treatment using a Turbo DNA-free kit (Ambion/Life Technologies, Carlsbad, CA, USA), and the NAC-test was repeated. All samples were DNA-negative following the second DNase-digestion. Only one single sample was not subjected to the NAC-test repetition because of a very limited RNA amount. Since all other samples were proven to be free of DNA-contamination following the second DNase treatment, we concluded that the second DNase digestion was successful also for this last, limited sample.

Transcript levels of *Taar8b* (mm03025167_gH), *Thra* (mm00579691_m1), and *Thrb* (mm00437044_m1) were determined by RT-qPCR, using specific TaqMan fluorescent reporter probe assays with *Gapdh* (mm99999915_g1) serving as the calibrator house-keeping gene (all TaqMan Gene Expression Assays: Applied Biosystems/Life Technologies, Carlsbad, CA, USA). Briefly, 2 µg of total RNA were reverse transcribed into cDNA using a High Capacity cDNA Reverse Transcription kit (Applied Biosystems, Foster City, CA, USA), except for three thyroid samples with restricted RNA yield, where only 1 to 1.2 µg of RNA were available for reverse transcription. Thermocycling for quantification of the initial transcript levels was performed in 20 µL reactions using TaqMan Gene Expression Master Mix, TaqMan Gene Expression Assay (both from Applied Biosystems), 100 ng of the corresponding cDNA and a 7900HT Fast Real-Time PCR System (Applied Biosystems). Initial denaturation at 95 °C for 10 min was followed by 50 cycles of denaturation at 95 °C for 15 s, and annealing/elongation at 60 °C for 60 s. All samples were analyzed in triplicates except for one thyroid pool sample with limited RNA yield, which was analyzed in duplicate. For two further thyroid samples, the qPCR assays —although performed in triplicates—were restricted to the reference gene Gapdh and the target gene Taar8b due to limited sample amount. The number of biological samples ranged for analyzed tissues between eight and 13 (liver: *n* = 13; heart: *n* = 13; kidney: *n* = 13; cerebellum: *n* = 12; cortex: *n* = 12; hippocampus: *n* = 10; striatum: *n* = 12; thyroid: *n* = 8; olfactory bulb: *n* = 8). One of the thyroid samples comprised a mixture of equal amounts of RNA/cDNA from two male mice. Each tissue group included at least three male and five female mice. For the graphical display, the results are provided as Δ*C*_t_ values normalized to *Gapdh* mRNA levels determined in the same sample. All *C*_t_-values of technical replicates for all analyzed samples are provided as Table S3.

### 4.3. Cloning of TAAR8

All full-length *TAAR8* and control constructs were cloned into the eukaryotic expression vector pcDps and *N*-terminally tagged with a hemagglutinin (5'-YPYDVPDYA-3') epitope (HA) for functional assays and determination of cell surface expression. In order to enhance cell surface expression, TAAR8 was *N*-terminally fused (between the HA epitope and the receptor) with the first 20 amino acids of the bovine rhodopsin (Rho)-tag as previously described [6,7,43]. Plasmids were sequenced and verified with BigDye-terminator sequencing (PerkinElmer Inc., Waltham, MA, USA) using an automatic sequencer (ABI3710xl; Applied Biosystems).

### 4.4. Cell Culture and Transient Transfection

For determination of signal transduction properties (via the second messenger cAMP, inositol trisphosphate (IP)-3, and RhoA), HEK293 cells (human embryonic kidney cells) were cultured in Minimum Essential Medium Earle’s (MEM) (Biochrom AG, Berlin, Germany) supplemented with 5% FBS (Biochrom AG, Berlin, Germany) and non-essential amino acids (Biochrom AG, Berlin, Germany) at 37 °C with 5% CO_2_.

Cell surface expression studies were conducted in COS-7 cells (derived from an African Green Monkey SV40-transfected kidney fibroblast cell line), which are more robust facing various washing steps and were cultured in Dulbecco’s Modified Eagle Medium (DMEM) (Biochrom AG, Berlin, Germany) at 37 °C with 5% CO_2_ and supplemented with 5% FBS and 100 U/mL penicillin, 100 µg/mL streptomycin (Biochrom AG, Berlin, Germany) and 2 mM l-glutamine (Invitrogen, Carlsbad, CA, USA).

All functional assays were carried out with cells grown in 96 well plates (1.5 × 10^4^ cells per well) coated with poly-l-lysin (Biochrom AG, Berlin, Germany). Cell surface expression was determined in 48 well plates (3.75 × 10^4^ cells per well). Twenty-eight hours following seeding, receptor DNA was co-transfected with the respective reporter construct (Promega, Fitchburg, WI, USA). Transient transfection was performed in supplement-free MEM or DMEM, respectively, using Metafectene according to the manufacturer’s protocol (Biontex, Munich, Germany).

The receptor ligands for functional assays were purchased from Sigma–Aldrich (St. Louis, MO, USA) (β-phenethylamine (PEA); bovine TSH (bTSH)) and from Santa Cruz Biotechnology (Dallas, TX, USA) (3-iodothyronamine (3-T_1_AM)).

### 4.5. Determination of Cell Surface Expression

Cell surface expression of the different receptor constructs was determined by an enzyme-linked immunosorbent assay (ELISA). Therefore, COS-7 cells were transiently transfected (six replicates in three to six independent experiments) with *N*-terminally HA-tagged TAAR1, TAAR8, Taar8b, and the human β2-adrenergic receptor (ADRB2) as a positive control and mock (pcDps). Cells were fixed with 4% *para*-formaldehyde 48 h after transfection and blocked with 10% FCS overnight. Cells were then incubated with biotin-labeled anti-HA antibody (Roche, Basel, Switzerland) (1:200) and detected with horseradish peroxidase-labeled Streptavidin (BioLegend, London, UK) (1:2500). Color reaction was achieved by the addition of the substrate *o*-phenylendiamine (Sigma–Aldrich, St. Louis, MO, USA) dissolved in a buffer composed of 0.1 M citric acid and 0.1 M sodium hydrogenphosphate enriched with hydrogen peroxide. The reactions were stopped by sodium sulfite that was over-saturated with 1 M hydrogen chloride. Absorption was measured at 492 and 620 nm by an Anthos Reader 2001 (Anthos Labtech Instruments, Salzburg, Austria).

### 4.6. Determination of G_s_ and G_i/o_ Activation by cAMP Accumulation Assay

G_s_ and G_i/o_ signaling properties were determined by a competitive cAMP accumulation assay based on AlphaScreen technology (Perkin–Elmer Life Science, Boston, MA, USA) as previously described [44,45]. HEK293 cells transiently transfected with the TAAR8 orthologues were incubated for 40 min with 10 µM ligand (3-T_1_AM or PEA). For measurement of G_i/o_ activation, cells were prestimulated with 50 µM forskolin (FSK) (Applichem, Darmstadt, Germany) or co-stimulated with 50 µM FSK and 10 µM of the respective ligand. Based on our own and previously published data, TAAR1 served as an assay control [9,16] for G_s_, and the human 5-hydroxytryptamine (serotonin) receptor 1B (5HT_1B_) as a positive controls for G_i/o_ activation [46]. Additionally, we tested for pertussis toxin (PTX) sensitive signaling by pre-incubating the cells with 50 µM PTX (Sigma–Aldrich, St. Louis, MO, USA) 20 h prior to ligand stimulation.

### 4.7. Determination of G_q/11_ and G_12/13_ Activation by Reporter Gene Assay

G_q/11_ and G_12/13_ activities were determined by luciferase reporter gene assay (IP3–luc or RhoA–luc), with the human thyrotropin receptor (TSHR) serving as a positive control [47]. HEK293 cells were co-transfected with TAAR8 or Taar8b and a reporter construct containing a responsive element and the firefly luciferase gene under the control of NFAT (nuclear factor of activated T-cells) in the case of G_q/11_ determination, or under the control of a serum response factor (SRF-RE) in the case of G_12/13_ determination. Two days post-transfection, cells were washed with PBS (Biochrom AG, Berlin, Germany) and stimulated for 6 h with 10 µM 3-T_1_AM or 10 µM PEA in supplement-free MEM at 37 °C with 5% CO_2_ saturated air. Reactions were terminated by aspirating the medium. Cells were lysed and then analyzed using the automatic luciferase substrate injection in accordance to the manufacturer’s protocols (Promega, Fitchburg, WI, USA), followed by quantification using a Berthold Microplate Reader (Berthold Technologies GmbH & Co. KG, Bad Wildbad, Germany). The human thyrotropin receptor (TSHR) served as a positive assay control [47].

### 4.8. Statistical Data Analysis

Results of functional data are indicated either as raw data or as fold over mock transfected. Basal state is shown as fold over basal mock transfection, and ligand stimulated results are referred to fold over ligand-stimulated mock with the respective substances in order to omit endogenous effects. Bar graphs and concentration response curves in which mean ± SEM are provided, and statistical analyses were generated using GraphPad Prism Version 6.0 (GraphPad Software, San Diego, CA, USA).

## 5. Conclusions

In summary, we analyzed the expression profile of *Taar8b* in different tissues by RT-qPCR and demonstrated *Taar8b* expression at most marginal expression levels, and no gender-specific differences were detected. Complete absence of expression could be validated for heart tissue using LNA *in situ* hybridization, thereby confirming previously published results. The physiological function of Taar8b in these tissues remains to be elucidated, particularly in combination with the yet unknown ligand. Moreover, for the first time, we confirmed basal G_i/o_ signaling activity for TAAR8 and Taar8b. However, 3-T_1_AM was not observed to induce detectable signaling of the TAAR8 subtype analyzed here and therefore, may not account for the ligand induced *in vivo* effects of 3-T_1_AM. It will be of future interest to search for other targets of 3-T_1_AM, including further known Taar8 subtypes or aminergic receptors.

## Supplementary Materials

Supplementary Tables can be found at http://www.mdpi.com/1422-0067/15/11/20638/s1.

## References

[1] Grandy D.K. (2007). Trace amine-associated receptor 1-family archetype or iconoclast?. Pharmacol. Ther..

[2] Zucchi R., Chiellini G., Scanlan T.S., Grandy D.K. (2006). Trace amine-associated receptors and their ligands. Br. J. Pharmacol..

[3] Lindemann L., Ebeling M., Kratochwil N.A., Bunzow J.R., Grandy D.K., Hoener M.C. (2005). Trace amine-associated receptors form structurally and functionally distinct subfamilies of novel G protein-coupled receptors. Genomics.

[4] Hussain A., Saraiva L.R., Korsching S.I. (2009). Positive Darwinian selection and the birth of an olfactory receptor clade in teleosts. Proc. Natl. Acad. Sci. USA.

[5] Tessarolo J.A., Tabesh M.J., Nesbitt M., Davidson W.S. (2014). Genomic organization and evolution of the trace amine-associated receptor (TAAR) repertoire in Atlantic salmon (*Salmo salar*). G3.

[6] Staubert C., Boselt I., Bohnekamp J., Rompler H., Enard W., Schoneberg T. (2010). Structural and functional evolution of the trace amine-associated receptors TAAR3, TAAR4 and TAAR5 in primates. PLoS One.

[7] Liberles S.D., Buck L.B. (2006). A second class of chemosensory receptors in the olfactory epithelium. Nature.

[8] Ferrero D.M., Wacker D., Roque M.A., Baldwin M.W., Stevens R.C., Liberles S.D. (2012). Agonists for 13 trace amine-associated receptors provide insight into the molecular basis of odor selectivity. ACS Chem. Biol..

[9] Scanlan T.S., Suchland K.L., Hart M.E., Chiellini G., Huang Y., Kruzich P.J., Frascarelli S., Crossley D.A., Bunzow J.R., Ronca-Testoni S. (2004). 3-Iodothyronamine is an endogenous and rapid-acting derivative of thyroid hormone. Nat. Med..

[10] Chiellini G., Frascarelli S., Ghelardoni S., Carnicelli V., Tobias S.C., DeBarber A., Brogioni S., Ronca-Testoni S., Cerbai E., Grandy D.K. (2007). Cardiac effects of 3-iodothyronamine: A new aminergic system modulating cardiac function. FASEB J..

[11] Vanti W.B., Nguyen T., Cheng R., Lynch K.R., George S.R., O’Dowd B.F. (2003). Novel human G-protein-coupled receptors. Biochem. Biophys. Res. Commun..

[12] Panas M.W., Xie Z., Panas H.N., Hoener M.C., Vallender E.J., Miller G.M. (2012). Trace amine associated receptor 1 signaling in activated lymphocytes. J. Neuroimmune Pharmacol..

[13] D’Andrea G., Terrazzino S., Fortin D., Farruggio A., Rinaldi L., Leon A. (2003). HPLC electrochemical detection of trace amines in human plasma and platelets and expression of mRNA transcripts of trace amine receptors in circulating leukocytes. Neurosci. Lett..

[14] Wasik A.M., Millan M.J., Scanlan T., Barnes N.M., Gordon J. (2012). Evidence for functional trace amine associated receptor-1 in normal and malignant B cells. Leuk Res..

[15] Babusyte A., Kotthoff M., Fiedler J., Krautwurst D. (2013). Biogenic amines activate blood leukocytes via trace amine-associated receptors TAAR1 and TAAR2. J. Leukoc. Biol..

[16] Borowsky B., Adham N., Jones K.A., Raddatz R., Artymyshyn R., Ogozalek K.L., Durkin M.M., Lakhlani P.P., Bonini J.A., Pathirana S. (2001). Trace amines: Identification of a family of mammalian G protein-coupled receptors. Proc. Natl. Acad. Sci. USA.

[17] Liberles S.D. (2009). Trace amine-associated receptors are olfactory receptors in vertebrates. Ann. N. Y. Acad. Sci..

[18] Carnicelli V., Santoro A., Sellari-Franceschini S., Berrettini S., Zucchi R. (2010). Expression of trace amine-associated receptors in human nasal mucosa. Chemosens. Percept..

[19] Johnson M.A., Tsai L., Roy D.S., Valenzuela D.H., Mosley C., Magklara A., Lomvardas S., Liberles S.D., Barnea G. (2012). Neurons expressing trace amine-associated receptors project to discrete glomeruli and constitute an olfactory subsystem. Proc. Natl. Acad. Sci. USA.

[20] Pacifico R., Dewan A., Cawley D., Guo C., Bozza T. (2012). An olfactory subsystem that mediates high-sensitivity detection of volatile amines. Cell Rep..

[21] Zhang J., Pacifico R., Cawley D., Feinstein P., Bozza T. (2013). Ultrasensitive detection of amines by a trace amine-associated receptor. J. Neurosci..

[22] Frascarelli S., Ghelardoni S., Chiellini G., Galli E., Ronca F., Scanlan T.S., Zucchi R. (2011). Cardioprotective effect of 3-iodothyronamine in perfused rat heart subjected to ischemia and reperfusion. Cardiovasc. Drugs Ther..

[23] Doyle K.P., Suchland K.L., Ciesielski T.M., Lessov N.S., Grandy D.K., Scanlan T.S., Stenzel-Poore M.P. (2007). Novel thyroxine derivatives, thyronamine and 3-iodothyronamine, induce transient hypothermia and marked neuroprotection against stroke injury. Stroke.

[24] Panas H.N., Lynch L.J., Vallender E.J., Xie Z., Chen G.L., Lynn S.K., Scanlan T.S., Miller G.M. (2010). Normal thermoregulatory responses to 3-iodothyronamine, trace amines and amphetamine-like psychostimulants in trace amine associated receptor 1 knockout mice. J. Neurosci. Res..

[25] Lindemann L., Meyer C.A., Jeanneau K., Bradaia A., Ozmen L., Bluethmann H., Bettler B., Wettstein J.G., Borroni E., Moreau J.L. (2008). Trace amine-associated receptor 1 modulates dopaminergic activity. J. Pharmacol. Exp. Ther..

[26] Wallrabenstein I., Kuklan J., Weber L., Zborala S., Werner M., Altmuller J., Becker C., Schmidt A., Hatt H., Hummel T. (2013). Human trace amine-associated receptor TAAR5 can be activated by trimethylamine. PLoS One.

[27] Chiellini G., Erba P., Carnicelli V., Manfredi C., Frascarelli S., Ghelardoni S., Mariani G., Zucchi R. (2012). Distribution of exogenous [^125^I]-3-iodothyronamine in mouse *in vivo*: Relationship with trace amine-associated receptors. J. Endocrinol..

[28] D’Andrea G., D’Arrigo A., Facchinetti F., del Giudice E., Colavito D., Bernardini D., Leon A. (2012). Octopamine, unlike other trace amines, inhibits responses of astroglia-enriched cultures to lipopolysaccharide via a beta-adrenoreceptor-mediated mechanism. Neurosci. Lett..

[29] Regard J.B., Sato I.T., Coughlin S.R. (2008). Anatomical profiling of G protein-coupled receptor expression. Cell.

[30] (2014). Personal communication.

[31] Thiel G., Kaufmann A., Rossler O.G. (2013). G-protein-coupled designer receptors—New chemical-genetic tools for signal transduction research. Biol. Chem..

[32] Eason M.G., Kurose H., Holt B.D., Raymond J.R., Liggett S.B. (1992). Simultaneous coupling of alpha 2-adrenergic receptors to two G-proteins with opposing effects. Subtype-selective coupling of alpha 2C10, alpha 2C4, and alpha 2C2 adrenergic receptors to Gi and Gs. J. Biol. Chem..

[33] Xiao R.P., Zhu W., Zheng M., Cao C., Zhang Y., Lakatta E.G., Han Q. (2006). Subtype-specific alpha1- and beta-adrenoceptor signaling in the heart. Trends Pharmacol. Sci..

[34] Jones S.V., Heilman C.J., Brann M.R. (1991). Functional responses of cloned muscarinic receptors expressed in CHO-K1 cells. Mol. Pharmacol..

[35] Wess J. (1998). Molecular basis of receptor/G-protein-coupling selectivity. Pharmacol. Ther..

[36] Raymond J.R. (1995). Multiple mechanisms of receptor-G protein signaling specificity. Am. J. Physiol..

[37] Rozenfeld R., Devi L.A. (2011). Exploring a role for heteromerization in GPCR signalling specificity. Biochem. J..

[38] Levoye A., Dam J., Ayoub M.A., Guillaume J.L., Jockers R. (2006). Do orphan G-protein-coupled receptors have ligand-independent functions? New insights from receptor heterodimers. EMBO Rep..

[39] Kuner R., Kohr G., Grunewald S., Eisenhardt G., Bach A., Kornau H.C. (1999). Role of heteromer formation in GABAB receptor function. Science.

[40] Ng G.Y., Clark J., Coulombe N., Ethier N., Hebert T.E., Sullivan R., Kargman S., Chateauneuf A., Tsukamoto N., McDonald T. (1999). Identification of a GABAB receptor subunit, gb2, required for functional GABAB receptor activity. J. Biol. Chem..

[41] Jones K.A., Borowsky B., Tamm J.A., Craig D.A., Durkin M.M., Dai M., Yao W.J., Johnson M., Gunwaldsen C., Huang L.Y. (1998). GABA(B) receptors function as a heteromeric assembly of the subunits GABA(B)R1 and GABA(B)R2. Nature.

[42] Chomczynski P., Sacchi N. (1987). Single-step method of RNA isolation by acid guanidinium thiocyanate-phenol-chloroform extraction. Anal. Biochem..

[43] Zhuang H., Matsunami H. (2007). Synergism of accessory factors in functional expression of mammalian odorant receptors. J. Biol. Chem..

[44] Kleinau G., Pratzka J., Nurnberg D., Gruters A., Fuhrer-Sakel D., Krude H., Kohrle J., Schoneberg T., Biebermann H. (2011). Differential modulation of beta-adrenergic receptor signaling by trace amine-associated receptor 1 agonists. PLoS One.

[45] Piechowski C.L., Rediger A., Lagemann C., Muhlhaus J., Muller A., Pratzka J., Tarnow P., Gruters A., Krude H., Kleinau G. (2013). Inhibition of melanocortin-4 receptor dimerization by substitutions in intracellular loop 2. J. Mol. Endocrinol..

[46] Hamblin M.W., Metcalf M.A., McGuffin R.W., Karpells S. (1992). Molecular cloning and functional characterization of a human 5-HT1B serotonin receptor: A homologue of the rat 5-HT1B receptor with 5-HT1D-like pharmacological specificity. Biochem. Biophys. Res. Commun..

[47] Laugwitz K.L., Allgeier A., Offermanns S., Spicher K., van Sande J., Dumont J.E., Schultz G. (1996). The human thyrotropin receptor: A heptahelical receptor capable of stimulating members of all four G protein families. Proc. Natl. Acad. Sci. USA.

